# Formation and structural characterization of a europium(II) mono(scorpionate) complex and a sterically crowded pyraza­bole

**DOI:** 10.1107/S2056989017016498

**Published:** 2017-11-21

**Authors:** Phil Liebing, Marcel Kühling, Josef Takats, Liane Hilfert, Frank T. Edelmann

**Affiliations:** a ETH Zürich, Laboratorium für Anorganische Chemie, Vladimir-Prelog-Weg 2, 8093 Zürich, Switzerland; b Chemisches Institut der Otto-von-Guericke-Universität Magdeburg, Universitätsplatz 2, 39106 Magdeburg, Germany; c Department of Chemistry, University of Alberta, Edmonton, Alberta, AB, Canada, T6G 2G2

**Keywords:** crystal structure, europium, pyrazolylborate, scorpionate, pyraza­bole

## Abstract

The reaction of EuI_2_(THF)_2_ with potassium hydro­tris­(3,5-diiso­propyl­pyrazol­yl)borate (K[HB(3,5-^*i*Pr2^pz)_3_] (= KTp^*i*Pr2^, pz = pyrazol­yl) in a molar ratio of 1:1.5 resulted in extensive ligand fragmentation and formation of the europium(II) mono(scorpionate) complex bis­(3,5-diisopropyl-1*H*-pyrazole)[hydro­tris­(3,5-diiso­propyl­pyrazol­yl)borato]iodido­europium(II), [Eu(C_27_H_46_BN_6_)I(C_9_H_16_N_2_)_2_] or (Tp^*i*Pr2^)(3,5-^*i*Pr2^pzH)_2_Eu^II^I, **1**, in high yield (78%). As a typical by-product, small amounts of the sterically crowded pyraza­bole derivative *trans*-4,8-bis­(3,5-diiso­propyl­pyrazol-1-yl)-1,3,5,7-tetra­iso­propyl­pyraza­bole, C_36_H_62_B_2_H_8_ or *trans*-{(3,5-^*i*Pr2^pz)HB(μ-3,5-^*i*Pr2^pz)}_2_, **2**, were formed. Both title compounds have been structurally characterized through single-crystal X-ray diffraction. In **1**, two isopropyl groups are each disordered over two orientations with occupancy ratios of 0.574 (10):0.426 (10) and 0.719 (16):0.281 (16). In **2**, one isopropyl group is similarly disordered, occupancy ratio 0.649 (9):0.351 (9).

## Abstract 2

Reaction of EuI_2_(THF)_2_ with K[HB(3,5-^*i*Pr2^pz)] (= KTp^*i*Pr2^, pz = pyrazol­yl) afforded the new europium(II) scorpionate complex (KTp^*i*Pr2^)(3,5-^*i*Pr2^pzH)_2_Eu^II^I (**1**) in addition to the sterically crowded pyraza­bole derivative *trans*-{(3,5-^*i*Pr2^pz)HB(μ-3,5-^*i*Pr2^pz)}_2_ (**2**) which were both structurally characterized through X-ray diffraction.

## Chemical context   

**Figure 1:**
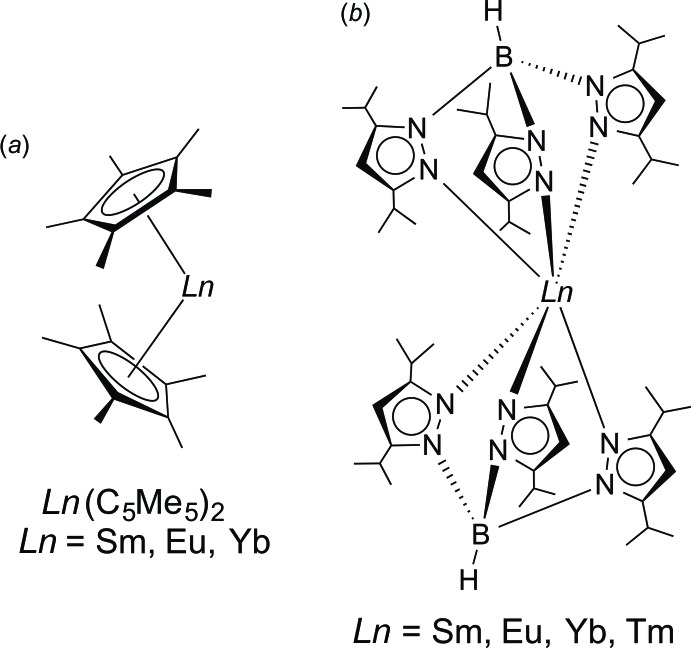
Comparison of the mol­ecular structures of ‘bent sandwich’-like lanthanide(II) cyclo­penta­dienides (*a*) and tris­(3,5-diiso­propyl­pyrazol­yl)borates (*b*).

The organometallic chemistry of divalent lanthanides provides fascinating structures such as the sandwich complexes *Ln*(C_5_Me_5_)_2_ (*Ln* = Sm, Eu, Yb; C_5_Me_5_ = *η* ^5^-penta­methyl­cyclo­penta­dien­yl). An unusual structural feature of the unsolvated lanthanide sandwich complexes *Ln*(C_5_Me_5_)_2_ (Fig. 1 ▸ *a*, *Ln* = Sm, Eu, Yb) is their bent metallocene structure in the solid state. This opens up the coordination sphere around the central divalent lanthanide ions and accounts for the very high reactivity of these compounds (Evans *et al.*, 1983 ▸, 1988 ▸; Evans, 2007 ▸). It has been demonstrated in the past that Trofimenko’s famous hydro­tris­(pyrazol­yl)borate ligands (‘scorpionates’) represent useful alternatives to the ubiquitous cyclo­penta­dienyl ligands (Pettinari, 2008 ▸; Trofimenko, 1966 ▸, 1993 ▸, 1999 ▸). Like the cyclo­penta­dienyl ligands, these trident­ate, monoanionic ligands can also be largely varied in their steric demand by introducing different substituents in the 3- and 5-positions of the pyrazolyl rings. According to Trofimenko’s nomenclature, the abbreviation Tp stands for the ring-unsubstituted hydro­tris­(pyrazol­yl)borate, whereas *e.g.* Tp^Me2^ denotes the sterically more demanding hydro­tris­(3,5-di­methyl­pyrazol­yl)borate. The homoleptic divalent lanthanide complexes *Ln*(Tp^Me2^)_2_ (*Ln* = Sm, Eu, Yb) have been found to adopt a highly symmetrical, trigonal–anti­prismatic coordination comprising an almost linear B⋯*Ln*⋯B arrangement (Marques *et al.*, 2002 ▸). Apparently, the sandwich-like structure of *Ln*(Tp^Me2^)_2_ is the result of the much larger cone angle of Tp^Me2^ (239°) as compared to that of the C_5_Me_5_ ligand (142°) (Davies *et al.*, 1985 ▸). More recently, these investigations have been successfully extended to the even larger hydro­tris­(3,5-diiso­propyl­pyrazol­yl)borate ligand (Tp^*i*Pr2^) (Kitajima *et al.*, 1992 ▸). Homoleptic complexes of this ligand could be isolated with the ‘classical’ divalent lanthanides samarium, europium, thulium and ytterbium (Momin *et al.*, 2014 ▸; Kühling *et al.*, 2015 ▸). Rather surprisingly, crystal structure determinations revealed a ‘bent sandwich’-like mol­ecular structure like *Ln*(C_5_Me_5_)_2_ (Fig. 1 ▸ *b*). Computational studies indicated that steric repulsion between the isopropyl groups forces the Tp^*i*Pr2^ ligands apart and permits the formation of unusual inter­ligand C—H⋯N hydrogen-bonding inter­actions that help to stabil­ize the structure (Momin *et al.*, 2014 ▸). The recently reported neon-yellow divalent europium complex Eu(Tp^*i*Pr2^)_2_ also stands out due to its bright-yellow photoluminescence, which has been investigated in great detail (Kühling *et al.*, 2015 ▸; Suta *et al.*, 2017 ▸). Eu(Tp^*i*Pr2^)_2_ was easily prepared in 83% yield by treatment of the bis-THF adduct of europium(II) diiodide, EuI_2_(THF)_2_, with 2 equiv. of KTp^*i*Pr2^ in THF solution (Kühling *et al.* 2015 ▸). We now report that the use of a significantly smaller amount of KTp^*i*Pr2^ led to extensive ligand fragmentation and formation of the first europium(II) mono(scorp­ion­ate) complex, [HB(3,5-^*iPr2*^pz)](3,5-^*i*Pr2^pzH)_2_Eu^II^I (**1**), in addition to a frequently observed by-product, the sterically crowded 4,8-bis­(pyrazol­yl)pyraza­bole derivative *trans*-{(3,5-^*i*Pr2^pz)HB(μ-3,5-^*i*Pr2^pz)}_2_ (**2**). Both products have been structurally characterized through single-crystal X-ray diffraction.

The starting material EuI_2_(THF)_2_ was prepared from Eu metal and 1,2-di­iodo­ethane using an established literature procedure (Girard *et al.*, 1980 ▸). The reaction of EuI_2_(THF)_2_ with 1.5 equiv. of KTp^*i*Pr2^ in THF produced a fluorescent, neon-yellow solution and a white precipitate of potassium iodide. Crystallization from *n*-pentane solvent afforded bright-yellow, air-sensitive crystals, which turned out to be the unexpected europium(II) mono(scorpionate) complex (Tp^*i*Pr2^)(3,5-^*i*Pr2^pzH)_2_Eu^II^I (**1**). The 78% isolated yield of **1** was surprisingly high. The coordinated neutral 3,5-diiso­propyl­yrazole ligands clearly resulted from fragmentation of the Tp^*i*Pr2^ ligand. *Ln*-induced fragmentation of substituted Tp ligands is well documented (Domingos *et al.*, 2002 ▸, and references cited therein), but it seems to be even more prevalent in the sterically highly demanding Tp^*i*Pr2^ system, as seen in some recently reported *Ln*(Tp^*i*Pr2^)-derived polysulfide complexes (Kühling *et al.*, 2016 ▸). Despite its paramagnetic nature, inter­pretable NMR spectra could be obtained for **1**. A single resonance at δ −5.3 ppm in the ^11^B NMR spectrum proved the presence of a single boron-containing species. A high-intensity peak at *m*/*z* 769 in the mass spectrum of **1** could be assigned to the fragment ion [Eu(Tp^*i*Pr2^)(^*i*Pr2^pz)]^+^, while a peak at *m*/*z* 616 corresponds to the ion [Eu(Tp^*i*Pr2^)]^+^.
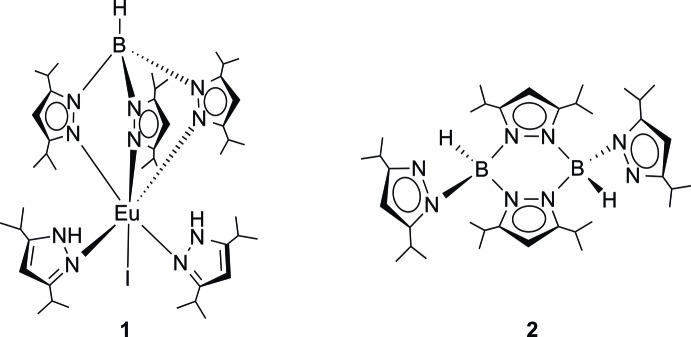


Further work-up of the supernatant solution remaining after isolation of **1** by addition of a large volume of non-polar hexa­methyl­disiloxane (HMDSO) resulted in the formation of well-formed, colorless, cube-like crystals in low yield. These turned out to be another ligand fragmentation product typical for lanthanide Tp chemistry, namely the 4,8-bis­(pyrazol­yl)pyraza­bole derivative *trans*-{(3,5-^*i*Pr2^pz)HB(μ-3,5-^*i*Pr2^pz)}_2_ (**2**). The parent pyraza­bole, {H_2_B(μ-pz)}_2_ has been known since 1966 when it was reported by Trofimenko contemporaneously with the discovery of Tp ligands (Trofimenko, 1966 ▸). Since then, numerous substituted pyraza­boles have been prepared and structurally investigated (Alcock & Sawyer, 1974 ▸; Cavero *et al.*, 2008 ▸; Niedenzu & Niedenzu, 1984 ▸; Niedenzu & Nöth, 1983 ▸; Trofimenko, 1966 ▸). In a number of recent studies, it has been demonstrated that certain substituted pyraza­boles possess unique photophysical and electrochemical properties and could thus find promising applications in organic photovoltaics (OPVs) and non-linear optics (Jadhav *et al.*, 2013 ▸, 2015 ▸; Misra *et al.*, 2013 ▸, 2014 ▸; Patil *et al.*, 2017 ▸). Compound **2** belongs to the rather special class of 4,8-bis­(pyrazol­yl)pyraza­boles in which two hydrogen atoms at boron are replaced by pyrazolyl moieties (Niedenzu & Niedenzu, 1984 ▸). Deliberate formation of the parent 4,8-bis­(pyrazol­yl)pyraza­bole, 4,8-*trans*-{(pz)HB(μ-pz)}_2_, has been achieved by thermolysis of the free acid of the hydro­tris­(pyrazol­yl)borate anion (Kresínski, 1999 ▸). In lanthanide Tp chemistry, such 4,8-(pyrazol­yl)pyraza­boles normally represent unwanted side-products as they frequently result from ligand fragmentation and are often the first crystalline products to come out of reaction mixtures (Kühling *et al.*, 2015 ▸, 2016 ▸; Lobbia *et al.*, 1992 ▸). Spectroscopic characterization of **2** was in good agreement with the results of the X-ray diffraction study. For instance, the mass spectrum of **2** showed the mol­ecular ion at *m*/*z* 627, and the ^11^B NMR spectrum displayed a single resonance at δ −4.3 ppm.

## Structural commentary   

**Figure 2:**
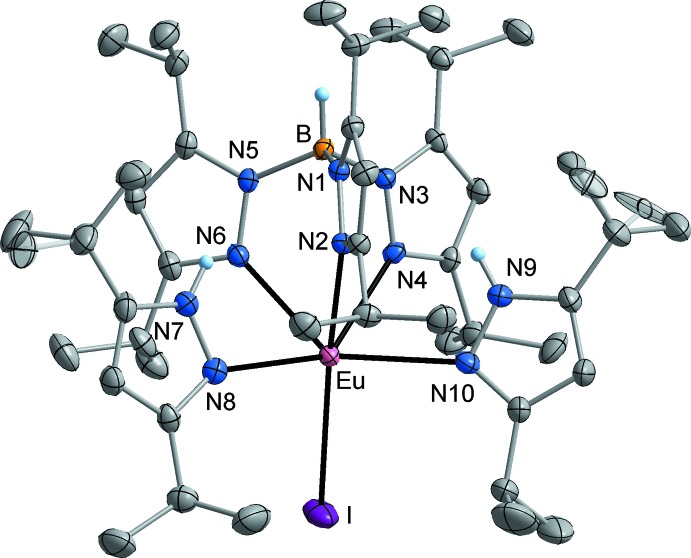
The mol­ecular structure of compound **1** in the crystal, showing orientational disorder of two isopropyl groups. Displacement ellipsoids are drawn at the 40% probability level, H atoms attached to C atoms omitted for clarity.

Both title compounds **1** and **2** exist as well-separated mol­ecules in the crystal. In the Eu^II^ complex **1**, one mol­ecule is present in the asymmetric unit (Fig. 2 ▸). The Tp^*i*Pr2^ ligand is attached to Eu in a symmetric tridentate mode with an H—B⋯Eu angle of 179.0 (2)°. The three Eu—N bonds cover the range 2.581 (2)–2.633 (2) Å, which resembles that observed in the corresponding homoleptic Eu^II^ complex Eu(Tp^*i*Pr2^)_2_ [2.563 (5)–2.670 (5) Å; Suta *et al.*, 2017 ▸]. The same applies to the B—N bonds, which are in the narrow range 1.547 (4)–1.555 (4) Å [Eu(Tp^*i*Pr2^)_2_: B—N = 1.531 (8)–1.559 (7) Å]. In **1**, the coordination of the iodido ligand relative to the (Tp^*i*Pr2^)^−^ ligand is slightly tilted [I—Eu⋯B = 151.49 (5)°], and an almost linear arrangement of the iodido ligand and one of the Tp^*i*Pr2^ N-donor atoms is realized [I—Eu—N2 = 165.92 (5)°]. A strongly distorted octa­hedral coordination is completed by the two neutral (3,5-^*i*Pr2^pzH) ligands, with coordination angles of 138.80 (7)° (N4—Eu—N8) and 137.43 (7)° (N6—Eu—N10). The corresponding Eu—N bond lengths [Eu—N8 = 2.699 (3), Eu—N10 = 2.660 (2) Å] are slightly longer than those to the (Tp^*i*Pr2^)^−^ ligand, which may be due to the absence of negative ligand charge. The NH⋯N distances between the two pyrazole NH moieties and potential hydrogen-acceptor atoms (N2, N4, N6) are in the range 2.512 (2)–2.610 (2) Å, but the groups are not in a proper orientation for efficient hydrogen bonding [N—H⋯N 115.0 (2)–122.0 (2)°]. Consequently, stabilization of the mol­ecular structure by intra­molecular hydrogen bonding is presumably of less importance.

**Figure 3:**
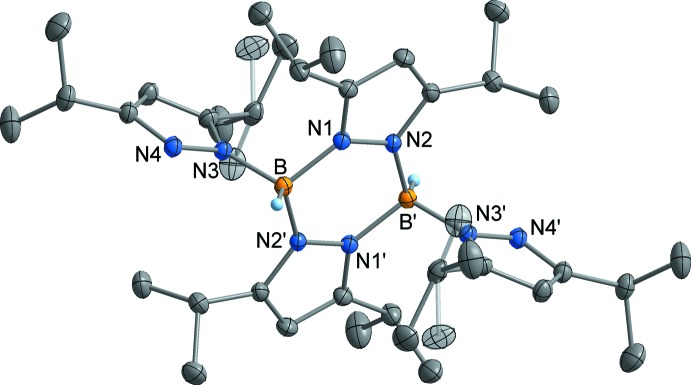
The mol­ecular structure of compound **2** in the crystal, showing orientational disorder of one isopropyl group. Displacement ellipsoids drawn at the 50% probability level, H atoms attached to C atoms omitted for clarity. [Symmetry code: (′) 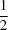 − *x*, 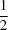 − *y*, −*z*.]

The pyraza­bol **2** exists as a centrosymmetric dimer in the crystal, which formally results from two HB(3,5-^*i*Pr2^pz)_2_ monomers (Fig. 3 ▸). The two B atoms are inter­connected by two μ-bridging (3,5-^*i*Pr2^pz) moieties, resulting in a planar, six-membered B_2_N_4_ ring. The B—N bonds within this ring are virtually equal at 1.554 (2) Å (B—N1) and 1.557 (2) Å (B—N2′), and therefore similar to that within the (Tp^*i*Pr2^)^−^ ligand in **1**. In contrast, the B—N bond to the terminal (3,5-^*i*Pr2^pz) moiety (B—N3) is slightly shortened to 1.532 (2) Å. The B atoms in **2** exhibit a virtually ideal tetra­hedral coordination with bonding angles in the narrow range 108.3 (1)–110.7 (1)°. The mol­ecular structure of **2** is very similar to that of the 3,5-di­methyl­pyrazolyl analog, *trans*-{(3,5-^Me2^pz)HB(μ-3,5-^Me2^pz)}_2_ [B—N = 1.5419 (2) and 1.5486 (1) Å for μ-(3,5-^Me2^pz) and 1.5257 (2) Å for terminal 3,5-^Me2^pz, N—B—N = 108.532 (6)–109.091 (6)°; Alcock & Sawyer, 1974 ▸]. In contrast, the unsubstituted pyraza­bol *trans*-{(pz)HB(μ-pz)}_2_ is non-centrosymmetric and features a remarkably puckered B_2_N_4_ ring [B—N = 1.546 (3)–1.559 (3) Å for μ-pz and 1.501 (3)–1.533 (3) Å for terminal pz, N—B—N = 105.2 (2)–111.0 (2)°; Kresiński, 1999 ▸].

## Supra­molecular features   

In both compounds **1** and **2**, no unusually short inter­molecular contacts have been observed. In **1**, the bulky ^*i*^Pr groups at the mol­ecule’s surface does not allow for inter­molecular N—H⋯N hydrogen bonding.

## Database survey   

For selected references on the reactivity of the sandwich complexes *Ln*(C_5_Me_5_)_2_ (*Ln* = Sm, Eu, Yb), see: Evans *et al.* (1983 ▸, 1988 ▸), Evans (2007 ▸).

For general information on scorpionate ligands, see: Kitajima *et al.* (1992 ▸), Pettinari (2008 ▸),Trofimenko (1966 ▸, 1999 ▸).

For the chemistry of divalent lanthanide scorpionate complexes, see: Davies *et al.* (1985 ▸), Domingos *et al.* (2002 ▸), Hillier *et al.* (2001 ▸), Kühling *et al.* (2015 ▸, 2016 ▸), Marques *et al.* (2002 ▸), Momin *et al.* (2014 ▸), Suta *et al.* (2017 ▸).

For general information on the chemistry and structures of pyraza­boles, see: Cavero *et al.* (2008 ▸), Niedenzu & Niedenzu (1984 ▸), Niedenzu & Nöth (1983 ▸), Trofimenko (1966 ▸).

For information on practical applications of pyraza­boles, see: Jadhav *et al.* (2013 ▸, 2015 ▸), Misra *et al.* (2013 ▸, 2014 ▸), Patil *et al.* (2017 ▸).

## Synthesis and crystallization   

All operations were performed under an argon atmosphere using standard Schlenk techniques. THF, hexa­methyl­disiloxane (HMDSO), and *n*-pentane were distilled from sodium/benzo­phenone under argon prior to use. NMR spectra were recorded on a Bruker DPX400 (^1^H: 400 MHz) spectrom­eter in THF-*D*_8_ at 295 (2) K. The ^11^B NMR spectra were obtained by using inverse gated decoupling on a Bruker Avance 400 NMR spectrometer, operating at 128.4 MHz. The external standard was 15 wt-% BF_3_·OEt_2_ in CDCl_3_ (δ_B_ = 0 ppm). IR spectra were measured on a Bruker Vertex V70 spectrometer equipped with a diamond ATR unit, electron impact mass spectra on a MAT95 spectrometer with an ioniz­ation energy of 70 eV. Elemental analyses (C, H and N) were performed using a VARIO EL cube apparatus. The starting materials EuI_2_(THF)_2_ (Girard *et al.* 1980 ▸) and KTp^*i*Pr2^ (Kitajima *et al.* 1992 ▸) were prepared according to published procedures.

Preparation of (Tp^*i*Pr2^)(3,5-^*i*Pr2^Hpz)_2_Eu^II^I (**1**) and *trans*-{(3,5-^*i*Pr2^pz)HB(μ-3,5-^*i*Pr2^pz)}_2_ (**2**): In a 250 mL Schlenk flask, THF (150 mL) was added to a mixture of EuI_2_(THF)_2_ (2.36 g, 4.29 mmol) and KTp^*i*Pr2^ (3.20 g, 6.33 mmol), and the resulting suspension was stirred for 12 h at r.t. A white precipitate (KI) was removed by filtration and the neon-yellow, fluorescent filtrate was evaporated to dryness. The residue was extracted with *n*-pentane (3 × 50 mL), the combined extracts filtered again and concentrated *in vacuo* to a total volume of *ca* 30 mL. Cooling to 277 K afforded bright-yellow, air-sensitive crystals of **1** (3.64 g, 78%), which were suitable for X-ray diffraction. The mother liquid was taken to dryness, and the slightly sticky residue was redissolved in *ca* 5 mL of THF. Addition of dry hexa­methyl­disiloxane (*ca* 50 mL) followed by cooling to 277 K for several days afforded *ca* 0.5 g of **2** as colorless, cube-like single-crystals.

**1**: Analysis calculated for C_45_H_78_BEuIN_10_, *M* = 1049.86 g mol^−1^: C 51.48, H 7.58, N 13.34%. Found: C 50.88, H 7.77, N 12.59%. M.p. *ca* 353 K (dec.). IR: *ν* 3173 *w*, 3096 *w* (*ν* C—H pyrazol­yl), 2961 *s*, 2929 *m*, 2869 *m* (ν CH_3_), 2550 *w* (νB—H), 1565 *w*, 1534 *m*, 1460 *s*, 1426 *m*, 1379 *s*, 1361 *s*, 1295 *m*, 1170 *vs*, 1104 *m*, 1046 *s*, 1012 *s*, 958 *w*, 923 *w*, 878 *w*, 787 *vs*, 767 *s*, 716 *m*, 659 *s*, 587 *w*, 511 *w*, 462 *w*, 389 *w*, 362 *w*, 306 *w*, 258 *w*, 219 *w*, 109 *s*, 75 *m* cm^−1. 1^H NMR (400.1 MHz, THF-*D_8_*, 300 K): *δ* 11.6 (*s br*, B—*H*), 5.70 (*s br*, 5H, C-*H* pyrazol­yl), 2.88 δ 153.8 (*br*, *q*-*C* pyrazol­yl), 98.7, 99.3 (*C*—H pyrazol­yl), 27.9, 32.1 (*C*—H ^*i*^Pr), 23.2 (*C*H_3_^*i*^Pr). ^11^B NMR (300 K, THF-*D_8_*, 128.4 MHz): δ −5.3 (*s, br*) ppm. MS: *m*/*z* (%) 769 (98) [Eu(Tp^*i*^Pr_2_)(^*i*Pr2^pz)]^+^, 616 (92) [Eu(Tp^*i*Pr2^)]^+^, 477 (85), 321 (100), 302 (55) [EuBH(^*i*Pr2^pz-CH_3_)]^+^, 152 (21) [^*i*Pr2^pz]^+^, 137 (63).

**2**: Analysis calculated for C_36_H_62_B_2_N_8_, *M* = 628.56 g mol^−1^: C 68.79, H 9.94, N 17.83%. Found: C 68.50, H 10.10, N 17.53%. M.p. 553 K. IR: *ν* 3176 *w*, 3094 *w* (*ν* C—H pyrazol­yl), 2966 *s*, 2928 *m*, 2869 *m*, 2825 *w* (*ν* CH_3_), 2467 *w* (*ν* B—H), 1576 *w*, 1541 *m*, 1497 *m*, 1461 *m*, 1369 *m*, 1301 *s*, 1233 *vs*, 1169 *vs*, 1134 *vs*, 1090 *s*, 1063 *s*, 1041 m, 1015 *m*, 982 *s*, 919 *w*, 879 *w*, 832 *s*, 788 *s*, 751 *m*, 723 *m*, 675 *m*, 566 *m*, 508 *m*, 473 *w*, 365 *m*, 302 *m*, 246 *m*, 137 *m*, 106 *m*, 75 *m* cm^−1. 1^H NMR (400.1 MHz, THF-*D_8_*, 300 K): δ 11.0 (*s br*, 2H, B—*H*), 5.75 (*s br*, 4H, C-*H* pyrazol­yl), 2.87–2.91 (*m*, 4H, C—*H ^i^*Pr), 1.15–1.27 (*m*, 48H, C*H*_3_^i^Pr) ppm. ^13^C NMR (300 K, THF-*D_8_*, 100 MHz): δ 160.7 (*br*, *q*-*C* pyrazol­yl), 97.5 (*C*—H pyrazol­yl), 28.5 (*C*—H ^*i*^Pr), 23.5 (*C*H_3_^*i*^Pr). ^11^B NMR (300 K, THF-*D_8_*, 128.4 MHz): δ −4.3 (*s, br*) ppm. MS: *m*/*z* (%) 627 (62) [*M*]^+^, 476 (100) [C_27_H_46_B_2_N_6_]^+^, 461 (75) [C_26_H_43_B_2_N_6_]^+^, 325 (66) [C_18_H_31_B_2_N_4_]^+^, 152 (74) [C_6_H_2_B_2_N_4_]^+^, 137 (89) [pz_2_].

## Refinement   

Crystal data, data collection and structure refinement details are summarized in Table 1 ▸. All H atoms were refined as riding atoms with B—H = 1.00 Å and C—H = 0.98–1.00 Å and *U*_iso_(H) = 1.5*U*_eq_(C) for methyl H atoms and *U*_iso_(H) = 1.2*U*_eq_(B,C) for all others. In **1**, two isopropyl groups are each disordered over two orientations with occupancy ratios of 0.574 (10):0.426 (10) and 0.719 (16):0.281 (16). In **2**, one isopropyl group is similarly disordered, occupancy ratio 0.649 (9):0.351 (9).

## Supplementary Material

Crystal structure: contains datablock(s) 1, 2. DOI: 10.1107/S2056989017016498/zl2718sup1.cif

Structure factors: contains datablock(s) 1. DOI: 10.1107/S2056989017016498/zl27181sup2.hkl

Structure factors: contains datablock(s) 2. DOI: 10.1107/S2056989017016498/zl27182sup3.hkl

CCDC references: 1585878, 1585877

Additional supporting information:  crystallographic information; 3D view; checkCIF report

## Figures and Tables

**Table 1:** Experimental details

	**1**	**2**
Crystal data		
Chemical formula	[Eu(C_27_H_46_BN_6_)I(C_9_H_16_N_2_)_2_]	C_36_H_62_B_2_N_8_
*M*_r_	1048.84	628.55
Crystal system, space group	Orthorhombic, *P* *b* *c* *a*	Monoclinic, *C*2/*c*
Temperature (K)	153	153
*a*, *b*, *c* (Å)	19.5319 (4), 26.6614 (4), 19.8681 (3)	25.7646 (11), 11.2134 (3), 15.0968 (7)
α, β, γ (°)	90, 90, 90	90, 118.792 (3), 90
*V* (Å^3^)	10346.3 (3)	3822.4 (3)
*Z*	8	4
Radiation type	Mo *K*α	Mo *K*α
μ (mm^−1^)	1.85	0.07
Crystal size (mm)	0.49 × 0.27 × 0.21	0.33 × 0.29 × 0.13

Data collection		
Diffractometer	Stoe IPDS 2T	Stoe IPDS 2T
Absorption correction	Numerical (*X-AREA* and *X-RED*; Stoe & Cie, 2002 ▸)	–
*T*_min_, *T*_max_	0.535, 0.716	–
No. of measured, independent and observed [*I* > 2σ(*I*)] reflections	43898, 10158, 8229	10509, 3367, 2594
*R*_int_	0.045	0.046
(sin θ/λ)_max_ (Å^−1^)	0.617	0.595

Refinement		
*R*[*F* ^2^ > 2σ(*F* ^2^)], *wR*(*F* ^2^), *S*	0.031, 0.063, 1.04	0.044, 0.104, 1.02
No. of reflections	10158	3367
No. of parameters	576	238
No. of restraints	24	0
H-atom treatment	H-atom parameters constrained	H-atom parameters constrained
Δρ_max_, Δρ_min_ (e Å^−3^)	1.24, −1.27	0.25, −0.21

Computer programs: *X-AREA* and *X-RED* (Stoe & Cie, 2002 ▸), *SIR97* (Altomare *et al.*, 1999 ▸), *SHELXL2016* (Sheldrick, 2015 ▸), *DIAMOND* (Brandenburg, 1999 ▸) and *publCIF* (Westrip, 2010 ▸).

## supplementary crystallographic information

### Bis(3,5-diisopropyl-1H-pyrazole)[hydrotris(3,5-diisopropylpyrazolyl)borato]iodidoeuropium(II) (1) Crystal data

**Table:**

[Eu(C_27_H_46_BN_6_)I(C_9_H_16_N_2_)_2_]	*D*_x_ = 1.347 Mg m^−^^3^
*M**_r_* = 1048.84	Mo *K*α radiation, λ = 0.71073 Å
Orthorhombic, *P**b**c**a*	Cell parameters from 44511 reflections
*a* = 19.5319 (4) Å	θ = 2.2–26.2°
*b* = 26.6614 (4) Å	µ = 1.85 mm^−^^1^
*c* = 19.8681 (3) Å	*T* = 153 K
*V* = 10346.3 (3) Å^3^	Block, yellow
*Z* = 8	0.49 × 0.27 × 0.21 mm
*F*(000) = 4312	

### Bis(3,5-diisopropyl-1H-pyrazole)[hydrotris(3,5-diisopropylpyrazolyl)borato]iodidoeuropium(II) (1) Data collection

**Table:**

Stoe IPDS 2T diffractometer	10158 independent reflections
Radiation source: fine-focus sealed tube	8229 reflections with *I* > 2σ(*I*)
Detector resolution: 6.67 pixels mm^-1^	*R*_int_ = 0.045
area detector scans	θ_max_ = 26.0°, θ_min_ = 2.2°
Absorption correction: numerical (X-AREA and X-RED; Stoe & Cie, 2002)	*h* = −23→24
*T*_min_ = 0.535, *T*_max_ = 0.716	*k* = −32→32
43898 measured reflections	*l* = −21→24

### Bis(3,5-diisopropyl-1H-pyrazole)[hydrotris(3,5-diisopropylpyrazolyl)borato]iodidoeuropium(II) (1) Refinement

**Table:**

Refinement on *F*^2^	Secondary atom site location: difference Fourier map
Least-squares matrix: full	Hydrogen site location: inferred from neighbouring sites
*R*[*F*^2^ > 2σ(*F*^2^)] = 0.031	H-atom parameters constrained
*wR*(*F*^2^) = 0.063	*w* = 1/[σ^2^(*F*_o_^2^) + (0.0226*P*)^2^ + 10.682*P*] where *P* = (*F*_o_^2^ + 2*F*_c_^2^)/3
*S* = 1.04	(Δ/σ)_max_ = 0.002
10158 reflections	Δρ_max_ = 1.24 e Å^−^^3^
576 parameters	Δρ_min_ = −1.27 e Å^−^^3^
24 restraints	Extinction correction: SHELXL2016 (Sheldrick, 2015), Fc^*^=kFc[1+0.001xFc^2^λ^3^/sin(2θ)]^-1/4^
Primary atom site location: heavy-atom method	Extinction coefficient: 0.00019 (2)

### Bis(3,5-diisopropyl-1H-pyrazole)[hydrotris(3,5-diisopropylpyrazolyl)borato]iodidoeuropium(II) (1) Special details

**Table:**

Geometry. All esds (except the esd in the dihedral angle between two l.s. planes) are estimated using the full covariance matrix. The cell esds are taken into account individually in the estimation of esds in distances, angles and torsion angles; correlations between esds in cell parameters are only used when they are defined by crystal symmetry. An approximate (isotropic) treatment of cell esds is used for estimating esds involving l.s. planes.

### Bis(3,5-diisopropyl-1H-pyrazole)[hydrotris(3,5-diisopropylpyrazolyl)borato]iodidoeuropium(II) (1) Fractional atomic coordinates and isotropic or equivalent isotropic displacement parameters (Å^2^)

**Table:**

	*x*	*y*	*z*	*U*_iso_*/*U*_eq_	Occ. (<1)
C1	0.23921 (15)	0.19065 (10)	0.45330 (14)	0.0232 (6)	
C2	0.21068 (16)	0.19034 (11)	0.38902 (14)	0.0273 (6)	
H4	0.183276	0.215746	0.369057	0.033*	
C3	0.23046 (15)	0.14552 (10)	0.36038 (14)	0.0238 (6)	
C4	0.23355 (17)	0.23068 (11)	0.50638 (15)	0.0283 (6)	
H5	0.263908	0.221037	0.544789	0.034*	
C5	0.25808 (19)	0.28129 (12)	0.47934 (18)	0.0384 (8)	
H8	0.255417	0.306493	0.515143	0.058*	
H6	0.228981	0.291544	0.441589	0.058*	
H7	0.305575	0.278252	0.463953	0.058*	
C6	0.16048 (18)	0.23508 (12)	0.53306 (17)	0.0381 (8)	
H10	0.158795	0.260725	0.568352	0.057*	
H9	0.146011	0.202746	0.551724	0.057*	
H11	0.129735	0.244581	0.496224	0.057*	
C7	0.21582 (15)	0.12724 (11)	0.29037 (14)	0.0291 (6)	
H12	0.218647	0.089789	0.290276	0.035*	
C8	0.14350 (19)	0.14246 (15)	0.26872 (18)	0.0492 (9)	
H14	0.133731	0.128534	0.224096	0.074*	
H13	0.140294	0.179121	0.266970	0.074*	
H15	0.110206	0.129474	0.301242	0.074*	
C9	0.2686 (2)	0.14745 (12)	0.24108 (16)	0.0392 (8)	
H17	0.259015	0.134409	0.195949	0.059*	
H18	0.314471	0.136803	0.255155	0.059*	
H16	0.266440	0.184172	0.240395	0.059*	
C10	0.48118 (14)	0.09139 (11)	0.45682 (14)	0.0244 (6)	
C11	0.49553 (15)	0.07902 (11)	0.39050 (14)	0.0274 (6)	
H19	0.539399	0.077925	0.369911	0.033*	
C12	0.43383 (15)	0.06867 (10)	0.36050 (14)	0.0238 (6)	
C13	0.52990 (15)	0.10295 (12)	0.51323 (15)	0.0309 (7)	
H20	0.502309	0.114062	0.552928	0.037*	
C14	0.56970 (19)	0.05623 (13)	0.53359 (17)	0.0432 (8)	
H21	0.600301	0.064330	0.571137	0.065*	
H22	0.596805	0.044372	0.495250	0.065*	
H23	0.537614	0.029938	0.547469	0.065*	
C15	0.57849 (19)	0.14513 (14)	0.4948 (2)	0.0483 (9)	
H24	0.607370	0.153213	0.533668	0.073*	
H25	0.552076	0.174828	0.481785	0.073*	
H26	0.607449	0.134594	0.457093	0.073*	
C16	0.42087 (16)	0.04955 (11)	0.29062 (14)	0.0287 (6)	
H27	0.374654	0.061415	0.275938	0.034*	
C17	0.4208 (2)	−0.00760 (12)	0.29044 (17)	0.0436 (9)	
H30	0.412317	−0.019769	0.244642	0.065*	
H28	0.384677	−0.019880	0.320468	0.065*	
H29	0.465295	−0.019921	0.306087	0.065*	
C18	0.4739 (2)	0.06968 (17)	0.24131 (17)	0.0512 (10)	
H32	0.460857	0.060380	0.195346	0.077*	
H33	0.518819	0.055308	0.251868	0.077*	
H31	0.476153	0.106295	0.245001	0.077*	
C19	0.26615 (17)	0.00515 (11)	0.55876 (15)	0.0290 (6)	
C20	0.23614 (18)	−0.02982 (12)	0.51569 (17)	0.0341 (7)	
H34	0.213786	−0.060076	0.528125	0.041*	
C21	0.24547 (15)	−0.01156 (11)	0.45133 (16)	0.0268 (7)	
C22	0.27416 (19)	0.00427 (12)	0.63403 (15)	0.0362 (7)	
H35	0.283022	0.039493	0.648967	0.043*	
C23	0.3355 (2)	−0.02675 (15)	0.65418 (18)	0.0469 (9)	
H37	0.340200	−0.026421	0.703283	0.070*	
H38	0.376857	−0.012635	0.633662	0.070*	
H36	0.329249	−0.061345	0.638663	0.070*	
C24	0.2098 (2)	−0.01320 (16)	0.66876 (19)	0.0524 (10)	
H39	0.217036	−0.013151	0.717576	0.079*	
H41	0.198657	−0.047253	0.653800	0.079*	
H40	0.172026	0.009446	0.657489	0.079*	
C25	0.22093 (18)	−0.03350 (12)	0.38605 (16)	0.0346 (7)	
H42	0.258186	−0.029403	0.351965	0.041*	
C26	0.2065 (2)	−0.08964 (13)	0.3940 (2)	0.0525 (10)	
H45	0.194344	−0.103937	0.350169	0.079*	
H44	0.168549	−0.094462	0.425556	0.079*	
H43	0.247523	−0.106445	0.411353	0.079*	
C27	0.1578 (2)	−0.00645 (14)	0.36021 (19)	0.0471 (9)	
H47	0.143055	−0.021654	0.317693	0.071*	
H46	0.168614	0.029013	0.352819	0.071*	
H48	0.120884	−0.009254	0.393429	0.071*	
C28	0.17695 (16)	0.13410 (11)	0.67075 (15)	0.0291 (6)	
C29	0.10972 (16)	0.11984 (13)	0.65687 (15)	0.0353 (7)	
H49	0.071667	0.121593	0.686550	0.042*	
C30	0.10960 (17)	0.10284 (13)	0.59193 (16)	0.0353 (7)	
C31	0.20606 (18)	0.15603 (13)	0.73387 (16)	0.0373 (8)	
H50	0.247595	0.136106	0.746193	0.045*	
C32	0.2284 (3)	0.20987 (16)	0.7225 (2)	0.0698 (13)	
H53	0.247710	0.223405	0.764241	0.105*	
H51	0.188727	0.230049	0.709049	0.105*	
H52	0.263059	0.210933	0.686909	0.105*	
C33	0.1558 (2)	0.15271 (18)	0.79216 (19)	0.0608 (11)	
H55	0.176796	0.167016	0.832654	0.091*	
H54	0.144132	0.117499	0.800382	0.091*	
H56	0.114222	0.171483	0.781002	0.091*	
C34A	0.0541 (2)	0.08120 (17)	0.54784 (19)	0.0532 (10)	0.574 (10)
H57A	0.013350	0.102080	0.559735	0.064*	0.574 (10)
C34B	0.0541 (2)	0.08120 (17)	0.54784 (19)	0.0532 (10)	0.426 (10)
H57B	0.066428	0.044801	0.548716	0.064*	0.426 (10)
C35	0.0648 (2)	0.09304 (16)	0.47511 (18)	0.0532 (10)	
H58	0.071183	0.129268	0.469644	0.080*	
H59	0.024658	0.082248	0.449277	0.080*	
H60	0.105487	0.075371	0.458703	0.080*	
C36A	0.0340 (5)	0.0322 (3)	0.5685 (5)	0.076 (3)	0.574 (10)
H61A	0.020303	0.033042	0.615986	0.114*	0.574 (10)
H62A	0.072501	0.009055	0.562895	0.114*	0.574 (10)
H63A	−0.004663	0.020941	0.541006	0.114*	0.574 (10)
C36B	−0.0104 (5)	0.0799 (5)	0.5750 (5)	0.063 (4)	0.426 (10)
H61B	−0.009018	0.062495	0.618433	0.095*	0.426 (10)
H62B	−0.041222	0.061930	0.544371	0.095*	0.426 (10)
H63B	−0.027094	0.114175	0.581517	0.095*	0.426 (10)
C37	0.43817 (16)	0.24760 (12)	0.54152 (15)	0.0315 (7)	
C38	0.46062 (18)	0.27479 (13)	0.48529 (16)	0.0387 (8)	
H64	0.484238	0.305937	0.485591	0.046*	
C39	0.44185 (17)	0.24765 (12)	0.43003 (15)	0.0341 (7)	
C40	0.4448 (2)	0.26136 (14)	0.61417 (16)	0.0458 (9)	
H65	0.422813	0.234499	0.641828	0.055*	
C41	0.5196 (3)	0.2646 (2)	0.6334 (2)	0.0818 (17)	
H67	0.523519	0.275708	0.680245	0.123*	
H68	0.542805	0.288635	0.603906	0.123*	
H66	0.540852	0.231495	0.628500	0.123*	
C42	0.4073 (3)	0.3109 (2)	0.6274 (2)	0.0861 (18)	
H69	0.412874	0.320390	0.674707	0.129*	
H71	0.358544	0.306816	0.617231	0.129*	
H70	0.426594	0.337220	0.598575	0.129*	
C43A	0.4504 (2)	0.25747 (16)	0.35604 (17)	0.0512 (10)	0.719 (16)
H72A	0.437857	0.293647	0.351016	0.061*	0.719 (16)
C43B	0.4504 (2)	0.25747 (16)	0.35604 (17)	0.0512 (10)	0.281 (16)
H72B	0.470887	0.291775	0.353444	0.061*	0.281 (16)
C44A	0.5189 (4)	0.2553 (5)	0.3336 (3)	0.072 (3)	0.719 (16)
H73A	0.521137	0.266047	0.286454	0.108*	0.719 (16)
H74A	0.535842	0.220884	0.337503	0.108*	0.719 (16)
H75A	0.547159	0.277643	0.361229	0.108*	0.719 (16)
C44B	0.5127 (12)	0.2193 (12)	0.3331 (8)	0.071 (6)	0.281 (16)
H73B	0.551346	0.222943	0.364141	0.106*	0.281 (16)
H74B	0.527507	0.227687	0.287342	0.106*	0.281 (16)
H75B	0.496165	0.184595	0.334190	0.106*	0.281 (16)
C45A	0.3975 (4)	0.2304 (5)	0.3138 (3)	0.057 (2)	0.719 (16)
H76A	0.351629	0.237583	0.331277	0.085*	0.719 (16)
H77A	0.405947	0.194154	0.315773	0.085*	0.719 (16)
H78A	0.400717	0.241788	0.267033	0.085*	0.719 (16)
C45B	0.3905 (11)	0.2610 (10)	0.3212 (9)	0.058 (5)	0.281 (16)
H76B	0.400386	0.264898	0.273114	0.087*	0.281 (16)
H77B	0.364638	0.290208	0.337140	0.087*	0.281 (16)
H78B	0.363373	0.230557	0.328252	0.087*	0.281 (16)
B	0.30572 (17)	0.06863 (11)	0.40118 (16)	0.0217 (6)	
H1	0.295416	0.053563	0.356212	0.026*	
N1	0.26882 (12)	0.12017 (8)	0.40630 (10)	0.0207 (4)	
N2	0.27389 (12)	0.14851 (8)	0.46446 (11)	0.0224 (5)	
N3	0.38405 (12)	0.07583 (8)	0.40739 (11)	0.0217 (5)	
N4	0.41373 (12)	0.09024 (9)	0.46732 (11)	0.0234 (5)	
N5	0.27948 (12)	0.03263 (8)	0.45666 (11)	0.0223 (5)	
N6	0.29237 (12)	0.04292 (9)	0.52350 (11)	0.0250 (5)	
N7	0.17398 (13)	0.10721 (9)	0.57009 (13)	0.0310 (5)	
H2	0.187129	0.098662	0.529310	0.037*	
N8	0.21714 (13)	0.12622 (9)	0.61732 (12)	0.0298 (5)	
N9	0.41090 (14)	0.20651 (10)	0.45416 (12)	0.0306 (6)	
H3	0.394258	0.182617	0.428346	0.037*	
N10	0.40781 (13)	0.20540 (10)	0.52242 (12)	0.0289 (5)	
I	0.42224 (2)	0.11967 (2)	0.71250 (2)	0.04898 (7)	
EU	0.34693 (2)	0.12375 (2)	0.57077 (2)	0.02286 (5)	

### Bis(3,5-diisopropyl-1H-pyrazole)[hydrotris(3,5-diisopropylpyrazolyl)borato]iodidoeuropium(II) (1) Atomic displacement parameters (Å^2^)

**Table:**

	*U*^11^	*U*^22^	*U*^33^	*U*^12^	*U*^13^	*U*^23^
C1	0.0221 (14)	0.0230 (14)	0.0245 (14)	0.0014 (12)	0.0007 (11)	−0.0010 (11)
C2	0.0259 (16)	0.0254 (14)	0.0305 (15)	0.0057 (12)	−0.0045 (12)	0.0012 (11)
C3	0.0214 (14)	0.0251 (13)	0.0248 (14)	0.0008 (12)	−0.0002 (11)	0.0017 (11)
C4	0.0319 (16)	0.0263 (14)	0.0268 (15)	0.0066 (13)	−0.0006 (13)	−0.0028 (12)
C5	0.044 (2)	0.0286 (16)	0.043 (2)	−0.0008 (15)	0.0017 (15)	−0.0063 (14)
C6	0.041 (2)	0.0350 (17)	0.0383 (18)	0.0086 (15)	0.0065 (15)	−0.0033 (13)
C7	0.0332 (16)	0.0271 (14)	0.0269 (14)	0.0015 (13)	−0.0069 (12)	−0.0031 (12)
C8	0.042 (2)	0.063 (2)	0.043 (2)	0.0119 (19)	−0.0176 (16)	−0.0166 (17)
C9	0.055 (2)	0.0339 (17)	0.0287 (16)	0.0040 (17)	0.0029 (16)	−0.0004 (13)
C10	0.0183 (14)	0.0261 (14)	0.0288 (14)	−0.0018 (12)	0.0004 (11)	0.0004 (11)
C11	0.0165 (14)	0.0387 (17)	0.0270 (15)	0.0004 (13)	0.0028 (11)	−0.0005 (12)
C12	0.0208 (15)	0.0276 (14)	0.0229 (13)	0.0021 (12)	0.0041 (11)	0.0026 (11)
C13	0.0218 (15)	0.0392 (16)	0.0316 (16)	−0.0052 (13)	−0.0003 (12)	−0.0060 (13)
C14	0.042 (2)	0.050 (2)	0.0380 (19)	−0.0024 (17)	−0.0161 (15)	0.0061 (15)
C15	0.037 (2)	0.049 (2)	0.059 (2)	−0.0175 (17)	−0.0167 (17)	0.0002 (17)
C16	0.0233 (15)	0.0394 (16)	0.0235 (14)	0.0020 (13)	0.0004 (12)	0.0018 (12)
C17	0.056 (2)	0.0411 (18)	0.0332 (17)	0.0113 (17)	−0.0097 (16)	−0.0104 (14)
C18	0.042 (2)	0.085 (3)	0.0259 (17)	−0.007 (2)	0.0069 (15)	0.0005 (17)
C19	0.0272 (15)	0.0295 (15)	0.0304 (16)	0.0003 (13)	0.0055 (13)	0.0063 (12)
C20	0.0340 (17)	0.0284 (16)	0.0398 (18)	−0.0063 (14)	0.0072 (15)	0.0070 (13)
C21	0.0226 (16)	0.0230 (15)	0.0348 (17)	−0.0024 (12)	0.0034 (11)	0.0012 (12)
C22	0.043 (2)	0.0365 (17)	0.0291 (16)	0.0030 (16)	0.0084 (14)	0.0101 (13)
C23	0.045 (2)	0.059 (2)	0.0371 (19)	0.0125 (19)	0.0034 (16)	0.0110 (16)
C24	0.045 (2)	0.072 (3)	0.041 (2)	0.005 (2)	0.0151 (17)	0.0221 (18)
C25	0.0351 (19)	0.0308 (16)	0.0378 (17)	−0.0121 (14)	0.0035 (14)	−0.0043 (13)
C26	0.068 (3)	0.0303 (18)	0.060 (2)	−0.0145 (19)	0.000 (2)	−0.0075 (16)
C27	0.044 (2)	0.048 (2)	0.050 (2)	−0.0111 (18)	−0.0107 (17)	−0.0014 (16)
C28	0.0284 (15)	0.0262 (15)	0.0328 (15)	0.0022 (12)	0.0073 (12)	0.0018 (12)
C29	0.0278 (16)	0.0426 (18)	0.0355 (16)	−0.0032 (15)	0.0066 (13)	0.0021 (14)
C30	0.0268 (17)	0.0393 (17)	0.0398 (17)	−0.0073 (14)	0.0024 (13)	0.0068 (14)
C31	0.0373 (19)	0.0404 (18)	0.0342 (17)	0.0038 (15)	0.0001 (14)	−0.0040 (14)
C32	0.098 (4)	0.056 (3)	0.055 (3)	−0.024 (3)	−0.013 (3)	−0.012 (2)
C33	0.060 (3)	0.086 (3)	0.037 (2)	−0.006 (2)	0.0109 (19)	−0.018 (2)
C34A	0.039 (2)	0.073 (3)	0.047 (2)	−0.025 (2)	−0.0084 (17)	0.0071 (19)
C34B	0.039 (2)	0.073 (3)	0.047 (2)	−0.025 (2)	−0.0084 (17)	0.0071 (19)
C35	0.048 (2)	0.067 (3)	0.045 (2)	−0.006 (2)	−0.0090 (17)	−0.0075 (18)
C36A	0.070 (6)	0.064 (5)	0.095 (6)	−0.041 (5)	−0.037 (5)	0.019 (5)
C36B	0.025 (5)	0.103 (9)	0.061 (6)	−0.020 (5)	0.011 (4)	−0.009 (6)
C37	0.0315 (17)	0.0348 (16)	0.0281 (15)	−0.0101 (14)	0.0032 (13)	−0.0003 (12)
C38	0.045 (2)	0.0397 (18)	0.0311 (16)	−0.0199 (16)	0.0014 (15)	0.0022 (13)
C39	0.0349 (17)	0.0413 (17)	0.0261 (15)	−0.0099 (15)	0.0018 (14)	0.0075 (14)
C40	0.055 (2)	0.057 (2)	0.0251 (16)	−0.0231 (19)	0.0060 (15)	−0.0063 (15)
C41	0.072 (3)	0.141 (5)	0.033 (2)	−0.041 (3)	−0.014 (2)	0.003 (3)
C42	0.125 (5)	0.085 (4)	0.048 (3)	−0.001 (3)	0.022 (3)	−0.027 (2)
C43A	0.064 (3)	0.065 (2)	0.0245 (16)	−0.022 (2)	0.0027 (17)	0.0075 (16)
C43B	0.064 (3)	0.065 (2)	0.0245 (16)	−0.022 (2)	0.0027 (17)	0.0075 (16)
C44A	0.058 (4)	0.128 (8)	0.031 (3)	−0.041 (5)	0.004 (3)	0.004 (4)
C44B	0.074 (11)	0.118 (14)	0.021 (6)	0.022 (11)	0.018 (7)	−0.003 (9)
C45A	0.054 (4)	0.093 (6)	0.023 (3)	−0.027 (4)	−0.005 (2)	0.007 (3)
C45B	0.081 (11)	0.062 (11)	0.031 (7)	−0.019 (10)	−0.007 (7)	0.017 (8)
B	0.0184 (16)	0.0228 (15)	0.0239 (15)	−0.0031 (13)	−0.0004 (12)	−0.0019 (12)
N1	0.0199 (11)	0.0201 (11)	0.0222 (10)	0.0001 (10)	0.0009 (9)	−0.0001 (9)
N2	0.0226 (12)	0.0242 (12)	0.0204 (11)	−0.0020 (10)	0.0010 (9)	−0.0024 (9)
N3	0.0205 (12)	0.0241 (11)	0.0204 (11)	−0.0007 (10)	0.0002 (9)	0.0006 (9)
N4	0.0180 (12)	0.0269 (12)	0.0252 (12)	−0.0016 (10)	−0.0020 (9)	−0.0040 (9)
N5	0.0189 (12)	0.0236 (11)	0.0245 (11)	−0.0014 (10)	0.0008 (9)	0.0005 (9)
N6	0.0231 (13)	0.0297 (12)	0.0222 (11)	−0.0009 (10)	0.0028 (9)	0.0022 (9)
N7	0.0296 (14)	0.0359 (13)	0.0274 (12)	−0.0050 (11)	0.0033 (11)	−0.0035 (11)
N8	0.0286 (13)	0.0304 (13)	0.0303 (12)	−0.0030 (12)	0.0006 (10)	−0.0002 (11)
N9	0.0353 (15)	0.0348 (14)	0.0216 (11)	−0.0072 (12)	−0.0006 (11)	−0.0016 (10)
N10	0.0300 (14)	0.0361 (14)	0.0206 (11)	−0.0020 (11)	−0.0001 (10)	0.0026 (10)
I	0.04194 (13)	0.07629 (18)	0.02871 (11)	0.00290 (13)	−0.00875 (9)	0.00910 (11)
EU	0.02157 (7)	0.02746 (7)	0.01955 (7)	−0.00247 (6)	0.00070 (5)	0.00068 (6)

### Bis(3,5-diisopropyl-1H-pyrazole)[hydrotris(3,5-diisopropylpyrazolyl)borato]iodidoeuropium(II) (1) Geometric parameters (Å, º)

**Table:**

C1—N2	1.331 (4)	C30—N7	1.336 (4)
C1—C2	1.393 (4)	C30—C34A	1.508 (5)
C1—C4	1.504 (4)	C30—C34B	1.508 (5)
C2—C3	1.379 (4)	C31—C32	1.517 (5)
C2—H4	0.9500	C31—C33	1.520 (5)
C3—N1	1.360 (3)	C31—H50	1.0000
C3—C7	1.501 (4)	C32—H53	0.9800
C4—C6	1.527 (4)	C32—H51	0.9800
C4—C5	1.529 (4)	C32—H52	0.9800
C4—H5	1.0000	C33—H55	0.9800
C5—H8	0.9800	C33—H54	0.9800
C5—H6	0.9800	C33—H56	0.9800
C5—H7	0.9800	C34a—C36A	1.424 (8)
C6—H10	0.9800	C34a—C35	1.494 (5)
C6—H9	0.9800	C34a—H57A	1.0000
C6—H11	0.9800	C34b—C36B	1.371 (9)
C7—C9	1.521 (4)	C34b—C35	1.494 (5)
C7—C8	1.531 (4)	C34b—H57B	1.0000
C7—H12	1.0000	C35—H58	0.9800
C8—H14	0.9800	C35—H59	0.9800
C8—H13	0.9800	C35—H60	0.9800
C8—H15	0.9800	C36a—H61A	0.9800
C9—H17	0.9800	C36a—H62A	0.9800
C9—H18	0.9800	C36a—H63A	0.9800
C9—H16	0.9800	C36b—H61B	0.9800
C10—N4	1.334 (4)	C36b—H62B	0.9800
C10—C11	1.387 (4)	C36b—H63B	0.9800
C10—C13	1.502 (4)	C37—N10	1.327 (4)
C11—C12	1.372 (4)	C37—C38	1.402 (4)
C11—H19	0.9500	C37—C40	1.495 (4)
C12—N3	1.360 (4)	C38—C39	1.365 (4)
C12—C16	1.501 (4)	C38—H64	0.9500
C13—C15	1.516 (5)	C39—N9	1.341 (4)
C13—C14	1.523 (5)	C39—C43A	1.502 (4)
C13—H20	1.0000	C39—C43B	1.502 (4)
C14—H21	0.9800	C40—C41	1.511 (6)
C14—H22	0.9800	C40—C42	1.534 (6)
C14—H23	0.9800	C40—H65	1.0000
C15—H24	0.9800	C41—H67	0.9800
C15—H25	0.9800	C41—H68	0.9800
C15—H26	0.9800	C41—H66	0.9800
C16—C18	1.523 (4)	C42—H69	0.9800
C16—C17	1.524 (4)	C42—H71	0.9800
C16—H27	1.0000	C42—H70	0.9800
C17—H30	0.9800	C43a—C44A	1.411 (8)
C17—H28	0.9800	C43a—C45A	1.514 (8)
C17—H29	0.9800	C43a—H72A	1.0000
C18—H32	0.9800	C43b—C45B	1.36 (2)
C18—H33	0.9800	C43b—C44B	1.651 (19)
C18—H31	0.9800	C43b—H72B	1.0000
C19—N6	1.329 (4)	C44a—H73A	0.9800
C19—C20	1.395 (5)	C44a—H74A	0.9800
C19—C22	1.504 (4)	C44a—H75A	0.9800
C20—C21	1.380 (4)	C44b—H73B	0.9800
C20—H34	0.9500	C44b—H74B	0.9800
C21—N5	1.357 (4)	C44b—H75B	0.9800
C21—C25	1.501 (4)	C45a—H76A	0.9800
C22—C24	1.507 (5)	C45a—H77A	0.9800
C22—C23	1.510 (5)	C45a—H78A	0.9800
C22—H35	1.0000	C45b—H76B	0.9800
C23—H37	0.9800	C45b—H77B	0.9800
C23—H38	0.9800	C45b—H78B	0.9800
C23—H36	0.9800	B—N3	1.547 (4)
C24—H39	0.9800	B—N5	1.549 (4)
C24—H41	0.9800	B—N1	1.555 (4)
C24—H40	0.9800	B—H1	1.0000
C25—C27	1.518 (5)	N1—N2	1.384 (3)
C25—C26	1.531 (4)	N2—EU	2.633 (2)
C25—H42	1.0000	N3—N4	1.379 (3)
C26—H45	0.9800	N4—EU	2.593 (2)
C26—H44	0.9800	N5—N6	1.379 (3)
C26—H43	0.9800	N6—EU	2.581 (2)
C27—H47	0.9800	N7—N8	1.359 (3)
C27—H46	0.9800	N7—H2	0.8800
C27—H48	0.9800	N8—EU	2.699 (2)
C28—N8	1.337 (4)	N9—N10	1.358 (3)
C28—C29	1.395 (4)	N9—H3	0.8800
C28—C31	1.496 (4)	N10—EU	2.660 (2)
C29—C30	1.367 (4)	I—EU	3.1788 (2)
C29—H49	0.9500		
			
N2—C1—C2	110.6 (2)	C31—C32—H52	109.5
N2—C1—C4	121.3 (3)	H53—C32—H52	109.5
C2—C1—C4	128.1 (3)	H51—C32—H52	109.5
C3—C2—C1	105.7 (3)	C31—C33—H55	109.5
C3—C2—H4	127.1	C31—C33—H54	109.5
C1—C2—H4	127.1	H55—C33—H54	109.5
N1—C3—C2	108.0 (2)	C31—C33—H56	109.5
N1—C3—C7	124.4 (2)	H55—C33—H56	109.5
C2—C3—C7	127.6 (3)	H54—C33—H56	109.5
C1—C4—C6	111.5 (3)	C36a—C34a—C35	120.8 (5)
C1—C4—C5	110.9 (3)	C36a—C34a—C30	112.4 (4)
C6—C4—C5	110.3 (3)	C35—C34a—C30	112.4 (3)
C1—C4—H5	108.0	C36a—C34a—H57A	102.8
C6—C4—H5	108.0	C35—C34a—H57A	102.8
C5—C4—H5	108.0	C30—C34a—H57A	102.8
C4—C5—H8	109.5	C36b—C34b—C35	120.9 (6)
C4—C5—H6	109.5	C36b—C34b—C30	116.2 (5)
H8—C5—H6	109.5	C35—C34b—C30	112.4 (3)
C4—C5—H7	109.5	C36b—C34b—H57B	100.9
H8—C5—H7	109.5	C35—C34b—H57B	100.9
H6—C5—H7	109.5	C30—C34b—H57B	100.9
C4—C6—H10	109.5	C34a—C35—H58	109.5
C4—C6—H9	109.5	C34a—C35—H59	109.5
H10—C6—H9	109.5	H58—C35—H59	109.5
C4—C6—H11	109.5	C34a—C35—H60	109.5
H10—C6—H11	109.5	H58—C35—H60	109.5
H9—C6—H11	109.5	H59—C35—H60	109.5
C3—C7—C9	110.6 (2)	C34a—C36a—H61A	109.5
C3—C7—C8	110.5 (3)	C34a—C36a—H62A	109.5
C9—C7—C8	110.5 (3)	H61a—C36a—H62A	109.5
C3—C7—H12	108.4	C34a—C36a—H63A	109.5
C9—C7—H12	108.4	H61a—C36a—H63A	109.5
C8—C7—H12	108.4	H62a—C36a—H63A	109.5
C7—C8—H14	109.5	C34b—C36b—H61B	109.5
C7—C8—H13	109.5	C34b—C36b—H62B	109.5
H14—C8—H13	109.5	H61b—C36b—H62B	109.5
C7—C8—H15	109.5	C34b—C36b—H63B	109.5
H14—C8—H15	109.5	H61b—C36b—H63B	109.5
H13—C8—H15	109.5	H62b—C36b—H63B	109.5
C7—C9—H17	109.5	N10—C37—C38	110.5 (3)
C7—C9—H18	109.5	N10—C37—C40	121.5 (3)
H17—C9—H18	109.5	C38—C37—C40	128.0 (3)
C7—C9—H16	109.5	C39—C38—C37	106.4 (3)
H17—C9—H16	109.5	C39—C38—H64	126.8
H18—C9—H16	109.5	C37—C38—H64	126.8
N4—C10—C11	110.0 (2)	N9—C39—C38	105.5 (3)
N4—C10—C13	120.9 (3)	N9—C39—C43A	122.8 (3)
C11—C10—C13	129.0 (3)	C38—C39—C43A	131.7 (3)
C12—C11—C10	106.4 (3)	N9—C39—C43B	122.8 (3)
C12—C11—H19	126.8	C38—C39—C43B	131.7 (3)
C10—C11—H19	126.8	C37—C40—C41	110.0 (3)
N3—C12—C11	107.6 (2)	C37—C40—C42	109.6 (3)
N3—C12—C16	124.1 (3)	C41—C40—C42	111.7 (4)
C11—C12—C16	128.2 (3)	C37—C40—H65	108.5
C10—C13—C15	111.6 (3)	C41—C40—H65	108.5
C10—C13—C14	110.7 (3)	C42—C40—H65	108.5
C15—C13—C14	110.5 (3)	C40—C41—H67	109.5
C10—C13—H20	107.9	C40—C41—H68	109.5
C15—C13—H20	107.9	H67—C41—H68	109.5
C14—C13—H20	107.9	C40—C41—H66	109.5
C13—C14—H21	109.5	H67—C41—H66	109.5
C13—C14—H22	109.5	H68—C41—H66	109.5
H21—C14—H22	109.5	C40—C42—H69	109.5
C13—C14—H23	109.5	C40—C42—H71	109.5
H21—C14—H23	109.5	H69—C42—H71	109.5
H22—C14—H23	109.5	C40—C42—H70	109.5
C13—C15—H24	109.5	H69—C42—H70	109.5
C13—C15—H25	109.5	H71—C42—H70	109.5
H24—C15—H25	109.5	C44a—C43a—C39	114.0 (4)
C13—C15—H26	109.5	C44a—C43a—C45A	116.9 (5)
H24—C15—H26	109.5	C39—C43a—C45A	112.6 (4)
H25—C15—H26	109.5	C44a—C43a—H72A	103.8
C12—C16—C18	111.1 (3)	C39—C43a—H72A	103.8
C12—C16—C17	110.0 (2)	C45a—C43a—H72A	103.8
C18—C16—C17	110.6 (3)	C45b—C43b—C39	114.5 (8)
C12—C16—H27	108.3	C45b—C43b—C44B	122.4 (12)
C18—C16—H27	108.3	C39—C43b—C44B	104.1 (7)
C17—C16—H27	108.3	C45b—C43b—H72B	104.7
C16—C17—H30	109.5	C39—C43b—H72B	104.7
C16—C17—H28	109.5	C44b—C43b—H72B	104.7
H30—C17—H28	109.5	C43a—C44a—H73A	109.5
C16—C17—H29	109.5	C43a—C44a—H74A	109.5
H30—C17—H29	109.5	H73a—C44a—H74A	109.5
H28—C17—H29	109.5	C43a—C44a—H75A	109.5
C16—C18—H32	109.5	H73a—C44a—H75A	109.5
C16—C18—H33	109.5	H74a—C44a—H75A	109.5
H32—C18—H33	109.5	C43b—C44b—H73B	109.5
C16—C18—H31	109.5	C43b—C44b—H74B	109.5
H32—C18—H31	109.5	H73b—C44b—H74B	109.5
H33—C18—H31	109.5	C43b—C44b—H75B	109.5
N6—C19—C20	110.2 (3)	H73b—C44b—H75B	109.5
N6—C19—C22	119.7 (3)	H74b—C44b—H75B	109.5
C20—C19—C22	130.1 (3)	C43a—C45a—H76A	109.5
C21—C20—C19	106.1 (3)	C43a—C45a—H77A	109.5
C21—C20—H34	127.0	H76a—C45a—H77A	109.5
C19—C20—H34	127.0	C43a—C45a—H78A	109.5
N5—C21—C20	107.4 (3)	H76a—C45a—H78A	109.5
N5—C21—C25	124.2 (3)	H77a—C45a—H78A	109.5
C20—C21—C25	128.4 (3)	C43b—C45b—H76B	109.5
C19—C22—C24	111.9 (3)	C43b—C45b—H77B	109.5
C19—C22—C23	110.8 (3)	H76b—C45b—H77B	109.5
C24—C22—C23	111.8 (3)	C43b—C45b—H78B	109.5
C19—C22—H35	107.4	H76b—C45b—H78B	109.5
C24—C22—H35	107.4	H77b—C45b—H78B	109.5
C23—C22—H35	107.4	N3—B—N5	110.3 (2)
C22—C23—H37	109.5	N3—B—N1	110.1 (2)
C22—C23—H38	109.5	N5—B—N1	110.3 (2)
H37—C23—H38	109.5	N3—B—H1	108.7
C22—C23—H36	109.5	N5—B—H1	108.7
H37—C23—H36	109.5	N1—B—H1	108.7
H38—C23—H36	109.5	C3—N1—N2	109.2 (2)
C22—C24—H39	109.5	C3—N1—B	130.6 (2)
C22—C24—H41	109.5	N2—N1—B	120.2 (2)
H39—C24—H41	109.5	C1—N2—N1	106.6 (2)
C22—C24—H40	109.5	C1—N2—EU	128.40 (18)
H39—C24—H40	109.5	N1—N2—EU	124.87 (16)
H41—C24—H40	109.5	C12—N3—N4	109.3 (2)
C21—C25—C27	111.5 (3)	C12—N3—B	129.4 (2)
C21—C25—C26	110.5 (3)	N4—N3—B	121.3 (2)
C27—C25—C26	110.5 (3)	C10—N4—N3	106.6 (2)
C21—C25—H42	108.1	C10—N4—EU	127.79 (18)
C27—C25—H42	108.1	N3—N4—EU	124.67 (17)
C26—C25—H42	108.1	C21—N5—N6	109.7 (2)
C25—C26—H45	109.5	C21—N5—B	130.1 (2)
C25—C26—H44	109.5	N6—N5—B	120.1 (2)
H45—C26—H44	109.5	C19—N6—N5	106.6 (2)
C25—C26—H43	109.5	C19—N6—EU	126.86 (19)
H45—C26—H43	109.5	N5—N6—EU	126.29 (16)
H44—C26—H43	109.5	C30—N7—N8	113.1 (3)
C25—C27—H47	109.5	C30—N7—H2	123.5
C25—C27—H46	109.5	N8—N7—H2	123.5
H47—C27—H46	109.5	C28—N8—N7	104.0 (2)
C25—C27—H48	109.5	C28—N8—EU	145.8 (2)
H47—C27—H48	109.5	N7—N8—EU	109.68 (17)
H46—C27—H48	109.5	C39—N9—N10	113.3 (2)
N8—C28—C29	110.7 (3)	C39—N9—H3	123.4
N8—C28—C31	120.3 (3)	N10—N9—H3	123.4
C29—C28—C31	129.1 (3)	C37—N10—N9	104.3 (2)
C30—C29—C28	106.2 (3)	C37—N10—EU	142.21 (19)
C30—C29—H49	126.9	N9—N10—EU	113.47 (18)
C28—C29—H49	126.9	N6—EU—N4	68.39 (7)
N7—C30—C29	106.0 (3)	N6—EU—N2	72.16 (7)
N7—C30—C34A	121.4 (3)	N4—EU—N2	73.92 (7)
C29—C30—C34A	132.6 (3)	N6—EU—N10	137.43 (7)
N7—C30—C34B	121.4 (3)	N4—EU—N10	76.75 (7)
C29—C30—C34B	132.6 (3)	N2—EU—N10	75.37 (7)
C28—C31—C32	110.7 (3)	N6—EU—N8	75.95 (7)
C28—C31—C33	111.8 (3)	N4—EU—N8	138.80 (7)
C32—C31—C33	110.7 (3)	N2—EU—N8	76.11 (7)
C28—C31—H50	107.8	N10—EU—N8	121.58 (8)
C32—C31—H50	107.8	N6—EU—I	119.00 (5)
C33—C31—H50	107.8	N4—EU—I	117.23 (5)
C31—C32—H53	109.5	N2—EU—I	165.92 (5)
C31—C32—H51	109.5	N10—EU—I	98.11 (5)
H53—C32—H51	109.5	N8—EU—I	97.58 (5)
			
N2—C1—C2—C3	−0.7 (3)	N9—C39—C43b—C44b	−75.4 (13)
C4—C1—C2—C3	−179.8 (3)	C38—C39—C43b—C44b	105.1 (13)
C1—C2—C3—N1	0.4 (3)	C2—C3—N1—N2	0.0 (3)
C1—C2—C3—C7	−177.4 (3)	C7—C3—N1—N2	177.9 (3)
N2—C1—C4—C6	−111.6 (3)	C2—C3—N1—B	−178.4 (3)
C2—C1—C4—C6	67.4 (4)	C7—C3—N1—B	−0.5 (5)
N2—C1—C4—C5	125.1 (3)	N3—B—N1—C3	116.0 (3)
C2—C1—C4—C5	−55.9 (4)	N5—B—N1—C3	−121.9 (3)
N1—C3—C7—C9	−92.6 (3)	N3—B—N1—N2	−62.2 (3)
C2—C3—C7—C9	84.9 (4)	N5—B—N1—N2	59.8 (3)
N1—C3—C7—C8	144.6 (3)	C2—C1—N2—N1	0.7 (3)
C2—C3—C7—C8	−37.9 (4)	C4—C1—N2—N1	179.8 (3)
N4—C10—C11—C12	−1.7 (3)	C2—C1—N2—EU	176.38 (19)
C13—C10—C11—C12	175.9 (3)	C4—C1—N2—EU	−4.5 (4)
C10—C11—C12—N3	1.3 (3)	C3—N1—N2—C1	−0.4 (3)
C10—C11—C12—C16	−174.9 (3)	B—N1—N2—C1	178.2 (2)
N4—C10—C13—C15	−127.3 (3)	C3—N1—N2—EU	−176.29 (17)
C11—C10—C13—C15	55.3 (4)	B—N1—N2—EU	2.3 (3)
N4—C10—C13—C14	109.1 (3)	C11—C12—N3—N4	−0.6 (3)
C11—C10—C13—C14	−68.3 (4)	C16—C12—N3—N4	175.8 (2)
N3—C12—C16—C18	150.9 (3)	C11—C12—N3—B	−178.8 (3)
C11—C12—C16—C18	−33.5 (4)	C16—C12—N3—B	−2.4 (4)
N3—C12—C16—C17	−86.3 (4)	N5—B—N3—C12	125.1 (3)
C11—C12—C16—C17	89.4 (4)	N1—B—N3—C12	−112.9 (3)
N6—C19—C20—C21	0.2 (4)	N5—B—N3—N4	−52.9 (3)
C22—C19—C20—C21	−177.3 (3)	N1—B—N3—N4	69.1 (3)
C19—C20—C21—N5	−0.3 (4)	C11—C10—N4—N3	1.3 (3)
C19—C20—C21—C25	−177.6 (3)	C13—C10—N4—N3	−176.5 (2)
N6—C19—C22—C24	139.4 (3)	C11—C10—N4—EU	−168.18 (19)
C20—C19—C22—C24	−43.4 (5)	C13—C10—N4—EU	14.0 (4)
N6—C19—C22—C23	−95.2 (4)	C12—N3—N4—C10	−0.5 (3)
C20—C19—C22—C23	82.1 (5)	B—N3—N4—C10	177.9 (2)
N5—C21—C25—C27	−75.4 (4)	C12—N3—N4—EU	169.45 (17)
C20—C21—C25—C27	101.5 (4)	B—N3—N4—EU	−12.2 (3)
N5—C21—C25—C26	161.3 (3)	C20—C21—N5—N6	0.2 (3)
C20—C21—C25—C26	−21.8 (5)	C25—C21—N5—N6	177.7 (3)
N8—C28—C29—C30	−0.3 (4)	C20—C21—N5—B	177.7 (3)
C31—C28—C29—C30	179.0 (3)	C25—C21—N5—B	−4.8 (5)
C28—C29—C30—N7	0.1 (4)	N3—B—N5—C21	−121.5 (3)
C28—C29—C30—C34a	178.3 (4)	N1—B—N5—C21	116.6 (3)
C28—C29—C30—C34b	178.3 (4)	N3—B—N5—N6	55.8 (3)
N8—C28—C31—C32	66.9 (4)	N1—B—N5—N6	−66.1 (3)
C29—C28—C31—C32	−112.3 (4)	C20—C19—N6—N5	0.0 (3)
N8—C28—C31—C33	−169.1 (3)	C22—C19—N6—N5	177.7 (3)
C29—C28—C31—C33	11.6 (5)	C20—C19—N6—EU	174.9 (2)
N7—C30—C34a—C36a	107.3 (6)	C22—C19—N6—EU	−7.3 (4)
C29—C30—C34a—C36a	−70.6 (7)	C21—N5—N6—C19	−0.1 (3)
N7—C30—C34a—C35	−32.9 (5)	B—N5—N6—C19	−177.9 (2)
C29—C30—C34a—C35	149.1 (4)	C21—N5—N6—EU	−175.07 (18)
N7—C30—C34b—C36b	−178.2 (7)	B—N5—N6—EU	7.1 (3)
C29—C30—C34b—C36b	3.8 (9)	C29—C30—N7—N8	0.1 (4)
N7—C30—C34b—C35	−32.9 (5)	C34a—C30—N7—N8	−178.3 (3)
C29—C30—C34b—C35	149.1 (4)	C34b—C30—N7—N8	−178.3 (3)
N10—C37—C38—C39	0.8 (4)	C29—C28—N8—N7	0.4 (3)
C40—C37—C38—C39	−178.8 (4)	C31—C28—N8—N7	−179.0 (3)
C37—C38—C39—N9	−0.8 (4)	C29—C28—N8—EU	−170.2 (3)
C37—C38—C39—C43a	178.8 (4)	C31—C28—N8—EU	10.4 (5)
C37—C38—C39—C43b	178.8 (4)	C30—N7—N8—C28	−0.3 (3)
N10—C37—C40—C41	117.8 (4)	C30—N7—N8—EU	174.1 (2)
C38—C37—C40—C41	−62.6 (5)	C38—C39—N9—N10	0.6 (4)
N10—C37—C40—C42	−119.1 (4)	C43a—C39—N9—N10	−179.1 (3)
C38—C37—C40—C42	60.5 (5)	C43b—C39—N9—N10	−179.1 (3)
N9—C39—C43a—C44a	−111.9 (7)	C38—C37—N10—N9	−0.5 (4)
C38—C39—C43a—C44a	68.5 (8)	C40—C37—N10—N9	179.2 (3)
N9—C39—C43a—C45a	24.2 (7)	C38—C37—N10—EU	179.1 (2)
C38—C39—C43a—C45a	−155.3 (6)	C40—C37—N10—EU	−1.2 (6)
N9—C39—C43b—C45b	60.8 (14)	C39—N9—N10—C37	−0.1 (4)
C38—C39—C43b—C45b	−118.7 (13)	C39—N9—N10—EU	−179.8 (2)

### trans-4,8-Bis(3,5-diisopropylpyrazol-1-yl)-1,3,5,7-tetraisopropylpyrazabole (2) Crystal data

**Table:**

C_36_H_62_B_2_N_8_	*F*(000) = 1376
*M**_r_* = 628.55	*D*_x_ = 1.092 Mg m^−^^3^
Monoclinic, *C*2/*c*	Mo *K*α radiation, λ = 0.71073 Å
*a* = 25.7646 (11) Å	Cell parameters from 11890 reflections
*b* = 11.2134 (3) Å	θ = 2.0–26.2°
*c* = 15.0968 (7) Å	µ = 0.07 mm^−^^1^
β = 118.792 (3)°	*T* = 153 K
*V* = 3822.4 (3) Å^3^	Plate, colorless
*Z* = 4	0.33 × 0.29 × 0.13 mm

### trans-4,8-Bis(3,5-diisopropylpyrazol-1-yl)-1,3,5,7-tetraisopropylpyrazabole (2) Data collection

**Table:**

Stoe IPDS 2T diffractometer	2594 reflections with *I* > 2σ(*I*)
Radiation source: fine-focus sealed tube	*R*_int_ = 0.046
Detector resolution: 6.67 pixels mm^-1^	θ_max_ = 25.0°, θ_min_ = 2.0°
area detector scans	*h* = −30→29
10509 measured reflections	*k* = −12→13
3367 independent reflections	*l* = −17→17

### trans-4,8-Bis(3,5-diisopropylpyrazol-1-yl)-1,3,5,7-tetraisopropylpyrazabole (2) Refinement

**Table:**

Refinement on *F*^2^	Secondary atom site location: difference Fourier map
Least-squares matrix: full	Hydrogen site location: inferred from neighbouring sites
*R*[*F*^2^ > 2σ(*F*^2^)] = 0.044	H-atom parameters constrained
*wR*(*F*^2^) = 0.104	*w* = 1/[σ^2^(*F*_o_^2^) + (0.050*P*)^2^ + 1.3932*P*] where *P* = (*F*_o_^2^ + 2*F*_c_^2^)/3
*S* = 1.02	(Δ/σ)_max_ < 0.001
3367 reflections	Δρ_max_ = 0.25 e Å^−^^3^
238 parameters	Δρ_min_ = −0.20 e Å^−^^3^
0 restraints	Extinction correction: SHELXL2016 (Sheldrick, 2015), Fc^*^=kFc[1+0.001xFc^2^λ^3^/sin(2θ)]^-1/4^
Primary atom site location: structure-invariant direct methods	Extinction coefficient: 0.0022 (4)

### trans-4,8-Bis(3,5-diisopropylpyrazol-1-yl)-1,3,5,7-tetraisopropylpyrazabole (2) Special details

**Table:**

Geometry. All esds (except the esd in the dihedral angle between two l.s. planes) are estimated using the full covariance matrix. The cell esds are taken into account individually in the estimation of esds in distances, angles and torsion angles; correlations between esds in cell parameters are only used when they are defined by crystal symmetry. An approximate (isotropic) treatment of cell esds is used for estimating esds involving l.s. planes.

### trans-4,8-Bis(3,5-diisopropylpyrazol-1-yl)-1,3,5,7-tetraisopropylpyrazabole (2) Fractional atomic coordinates and isotropic or equivalent isotropic displacement parameters (Å^2^)

**Table:**

	*x*	*y*	*z*	*U*_iso_*/*U*_eq_	Occ. (<1)
C1	0.32091 (7)	0.43231 (14)	0.12232 (11)	0.0228 (3)	
C2	0.31092 (7)	0.42821 (15)	0.20414 (12)	0.0275 (4)	
H2	0.328176	0.478383	0.261967	0.033*	
C3	0.27098 (7)	0.33708 (14)	0.18577 (11)	0.0239 (3)	
C4	0.36140 (7)	0.51263 (14)	0.10426 (12)	0.0255 (4)	
H3	0.350866	0.506956	0.031400	0.031*	
C5	0.42566 (7)	0.47407 (17)	0.16846 (13)	0.0361 (4)	
H5	0.451450	0.527573	0.155810	0.054*	
H6	0.430455	0.392253	0.150739	0.054*	
H4	0.436389	0.477573	0.240128	0.054*	
C6	0.35282 (9)	0.64149 (16)	0.12723 (15)	0.0390 (4)	
H9	0.378721	0.693891	0.113901	0.059*	
H8	0.362781	0.648366	0.198397	0.059*	
H7	0.311453	0.664942	0.084152	0.059*	
C7	0.24394 (7)	0.29648 (16)	0.24900 (12)	0.0302 (4)	
H10	0.223957	0.218098	0.222317	0.036*	
C8	0.29206 (10)	0.2793 (2)	0.35783 (14)	0.0543 (6)	
H11	0.274068	0.253611	0.398945	0.081*	
H12	0.313065	0.354794	0.384237	0.081*	
H13	0.320074	0.218456	0.360467	0.081*	
C9	0.19746 (9)	0.38648 (18)	0.24156 (16)	0.0451 (5)	
H15	0.179127	0.358161	0.281449	0.068*	
H14	0.167006	0.395218	0.170735	0.068*	
H16	0.216435	0.463780	0.267740	0.068*	
C10	0.06813 (7)	0.23099 (14)	0.02850 (12)	0.0257 (4)	
C11	0.06359 (7)	0.32593 (15)	−0.03555 (13)	0.0290 (4)	
H17	0.031464	0.379970	−0.068315	0.035*	
C12	0.11522 (7)	0.32426 (14)	−0.04087 (11)	0.0242 (3)	
C13	0.02374 (7)	0.19290 (17)	0.06011 (13)	0.0322 (4)	
H18	0.039684	0.120355	0.103467	0.039*	
C14	0.01516 (10)	0.2889 (2)	0.12299 (18)	0.0527 (6)	
H20	−0.011435	0.259168	0.147452	0.079*	
H19	−0.002167	0.359966	0.081317	0.079*	
H21	0.053547	0.309303	0.180752	0.079*	
C15	−0.03477 (8)	0.1591 (2)	−0.03071 (16)	0.0520 (6)	
H24	−0.061559	0.126983	−0.007583	0.078*	
H23	−0.027873	0.098683	−0.070761	0.078*	
H22	−0.052662	0.229990	−0.072424	0.078*	
C16A	0.13485 (7)	0.40591 (15)	−0.09797 (13)	0.0292 (4)	0.649 (9)
H25A	0.168807	0.367438	−0.101562	0.035*	0.649 (9)
C17A	0.1569 (3)	0.5251 (3)	−0.0392 (4)	0.0435 (11)	0.649 (9)
H26A	0.171675	0.577049	−0.074376	0.065*	0.649 (9)
H27A	0.188876	0.508864	0.029262	0.065*	0.649 (9)
H28A	0.124079	0.564576	−0.035502	0.065*	0.649 (9)
C18A	0.0865 (2)	0.4274 (6)	−0.2035 (3)	0.0535 (14)	0.649 (9)
H29A	0.101968	0.474541	−0.240313	0.080*	0.649 (9)
H30A	0.054076	0.470950	−0.201581	0.080*	0.649 (9)
H31A	0.071760	0.350805	−0.237778	0.080*	0.649 (9)
C16B	0.13485 (7)	0.40591 (15)	−0.09797 (13)	0.0292 (4)	0.351 (9)
H25B	0.178789	0.399759	−0.068850	0.035*	0.351 (9)
C17B	0.1037 (4)	0.3646 (8)	−0.2133 (5)	0.045 (2)	0.351 (9)
H26B	0.115240	0.418278	−0.251917	0.067*	0.351 (9)
H27B	0.060562	0.366928	−0.241231	0.067*	0.351 (9)
H28B	0.116013	0.283030	−0.217459	0.067*	0.351 (9)
C18B	0.1187 (6)	0.5326 (6)	−0.0920 (9)	0.053 (3)	0.351 (9)
H29B	0.132204	0.583712	−0.129447	0.079*	0.351 (9)
H30B	0.137625	0.557807	−0.021063	0.079*	0.351 (9)
H31B	0.075580	0.539310	−0.121428	0.079*	0.351 (9)
B	0.21039 (7)	0.18853 (16)	0.04185 (12)	0.0209 (4)	
H1	0.220947	0.117181	0.086985	0.025*	
N1	0.25728 (5)	0.28776 (11)	0.09607 (9)	0.0205 (3)	
N2	0.28824 (5)	0.34639 (11)	0.05681 (9)	0.0198 (3)	
N3	0.14859 (5)	0.23196 (11)	0.01778 (9)	0.0207 (3)	
N4	0.11934 (6)	0.17386 (12)	0.06116 (9)	0.0235 (3)	

### trans-4,8-Bis(3,5-diisopropylpyrazol-1-yl)-1,3,5,7-tetraisopropylpyrazabole (2) Atomic displacement parameters (Å^2^)

**Table:**

	*U*^11^	*U*^22^	*U*^33^	*U*^12^	*U*^13^	*U*^23^
C1	0.0190 (8)	0.0221 (8)	0.0248 (8)	−0.0002 (6)	0.0085 (6)	−0.0021 (6)
C2	0.0270 (9)	0.0295 (9)	0.0254 (8)	−0.0041 (7)	0.0121 (7)	−0.0077 (7)
C3	0.0220 (8)	0.0275 (8)	0.0210 (7)	−0.0009 (7)	0.0095 (6)	−0.0027 (6)
C4	0.0244 (8)	0.0246 (8)	0.0256 (8)	−0.0043 (7)	0.0105 (7)	−0.0019 (7)
C5	0.0250 (9)	0.0431 (10)	0.0374 (10)	−0.0067 (8)	0.0128 (8)	−0.0010 (8)
C6	0.0451 (11)	0.0273 (9)	0.0485 (11)	−0.0060 (8)	0.0255 (10)	−0.0046 (8)
C7	0.0306 (9)	0.0372 (10)	0.0262 (8)	−0.0092 (8)	0.0165 (7)	−0.0063 (7)
C8	0.0474 (12)	0.0863 (17)	0.0293 (10)	−0.0183 (12)	0.0185 (9)	0.0022 (10)
C9	0.0496 (12)	0.0469 (12)	0.0550 (12)	−0.0104 (10)	0.0382 (10)	−0.0136 (10)
C10	0.0213 (8)	0.0295 (9)	0.0277 (8)	−0.0015 (7)	0.0130 (7)	−0.0006 (7)
C11	0.0234 (8)	0.0294 (9)	0.0350 (9)	0.0046 (7)	0.0147 (7)	0.0040 (7)
C12	0.0234 (8)	0.0229 (8)	0.0265 (8)	0.0023 (7)	0.0122 (7)	0.0009 (6)
C13	0.0262 (9)	0.0385 (10)	0.0368 (9)	−0.0005 (8)	0.0190 (8)	0.0045 (8)
C14	0.0529 (13)	0.0612 (14)	0.0649 (14)	−0.0020 (11)	0.0450 (12)	−0.0062 (11)
C15	0.0312 (10)	0.0748 (15)	0.0488 (12)	−0.0145 (10)	0.0184 (9)	0.0055 (11)
C16A	0.0271 (9)	0.0287 (9)	0.0360 (9)	0.0053 (7)	0.0183 (8)	0.0086 (7)
C17A	0.054 (3)	0.0287 (17)	0.056 (2)	−0.0048 (18)	0.033 (2)	0.0051 (16)
C18A	0.045 (2)	0.067 (3)	0.0401 (19)	−0.010 (2)	0.0142 (17)	0.020 (2)
C16B	0.0271 (9)	0.0287 (9)	0.0360 (9)	0.0053 (7)	0.0183 (8)	0.0086 (7)
C17B	0.048 (4)	0.053 (5)	0.036 (3)	0.001 (3)	0.022 (3)	0.012 (3)
C18B	0.079 (7)	0.030 (3)	0.075 (6)	0.009 (4)	0.058 (6)	0.013 (3)
B	0.0200 (9)	0.0204 (9)	0.0215 (8)	−0.0001 (7)	0.0093 (7)	0.0012 (7)
N1	0.0186 (6)	0.0228 (7)	0.0205 (6)	−0.0007 (5)	0.0098 (5)	−0.0005 (5)
N2	0.0167 (6)	0.0214 (7)	0.0215 (6)	−0.0011 (5)	0.0095 (5)	0.0001 (5)
N3	0.0199 (7)	0.0215 (7)	0.0226 (6)	−0.0008 (5)	0.0117 (5)	0.0009 (5)
N4	0.0224 (7)	0.0261 (7)	0.0250 (7)	−0.0028 (6)	0.0138 (6)	−0.0002 (5)

### trans-4,8-Bis(3,5-diisopropylpyrazol-1-yl)-1,3,5,7-tetraisopropylpyrazabole (2) Geometric parameters (Å, º)

**Table:**

C1—N2	1.3464 (19)	C13—C14	1.520 (3)
C1—C2	1.379 (2)	C13—H18	1.0000
C1—C4	1.499 (2)	C14—H20	0.9800
C2—C3	1.380 (2)	C14—H19	0.9800
C2—H2	0.9500	C14—H21	0.9800
C3—N1	1.3431 (19)	C15—H24	0.9800
C3—C7	1.498 (2)	C15—H23	0.9800
C4—C5	1.524 (2)	C15—H22	0.9800
C4—C6	1.527 (2)	C16a—C18A	1.496 (4)
C4—H3	1.0000	C16a—C17A	1.552 (4)
C5—H5	0.9800	C16a—H25A	1.0000
C5—H6	0.9800	C17a—H26A	0.9800
C5—H4	0.9800	C17a—H27A	0.9800
C6—H9	0.9800	C17a—H28A	0.9800
C6—H8	0.9800	C18a—H29A	0.9800
C6—H7	0.9800	C18a—H30A	0.9800
C7—C8	1.520 (3)	C18a—H31A	0.9800
C7—C9	1.528 (3)	C16b—C18B	1.496 (7)
C7—H10	1.0000	C16b—C17B	1.595 (7)
C8—H11	0.9800	C16b—H25B	1.0000
C8—H12	0.9800	C17b—H26B	0.9800
C8—H13	0.9800	C17b—H27B	0.9800
C9—H15	0.9800	C17b—H28B	0.9800
C9—H14	0.9800	C18b—H29B	0.9800
C9—H16	0.9800	C18b—H30B	0.9800
C10—N4	1.330 (2)	C18b—H31B	0.9800
C10—C11	1.405 (2)	B—N3	1.532 (2)
C10—C13	1.498 (2)	B—N1	1.554 (2)
C11—C12	1.371 (2)	B—N2^i^	1.557 (2)
C11—H17	0.9500	B—H1	1.0000
C12—N3	1.364 (2)	N1—N2	1.3693 (16)
C12—C16A	1.502 (2)	N2—B^i^	1.557 (2)
C12—C16B	1.502 (2)	N3—N4	1.3772 (17)
C13—C15	1.518 (3)		
			
N2—C1—C2	108.29 (13)	C13—C14—H19	109.5
N2—C1—C4	122.67 (13)	H20—C14—H19	109.5
C2—C1—C4	129.02 (14)	C13—C14—H21	109.5
C1—C2—C3	106.71 (14)	H20—C14—H21	109.5
C1—C2—H2	126.6	H19—C14—H21	109.5
C3—C2—H2	126.6	C13—C15—H24	109.5
N1—C3—C2	108.41 (13)	C13—C15—H23	109.5
N1—C3—C7	122.71 (14)	H24—C15—H23	109.5
C2—C3—C7	128.86 (14)	C13—C15—H22	109.5
C1—C4—C5	110.60 (13)	H24—C15—H22	109.5
C1—C4—C6	109.73 (13)	H23—C15—H22	109.5
C5—C4—C6	110.85 (14)	C18a—C16a—C12	111.80 (19)
C1—C4—H3	108.5	C18a—C16a—C17A	111.0 (3)
C5—C4—H3	108.5	C12—C16a—C17A	109.18 (17)
C6—C4—H3	108.5	C18a—C16a—H25A	108.3
C4—C5—H5	109.5	C12—C16a—H25A	108.3
C4—C5—H6	109.5	C17a—C16a—H25A	108.3
H5—C5—H6	109.5	C16a—C17a—H26A	109.5
C4—C5—H4	109.5	C16a—C17a—H27A	109.5
H5—C5—H4	109.5	H26a—C17a—H27A	109.5
H6—C5—H4	109.5	C16a—C17a—H28A	109.5
C4—C6—H9	109.5	H26a—C17a—H28A	109.5
C4—C6—H8	109.5	H27a—C17a—H28A	109.5
H9—C6—H8	109.5	C16a—C18a—H29A	109.5
C4—C6—H7	109.5	C16a—C18a—H30A	109.5
H9—C6—H7	109.5	H29a—C18a—H30A	109.5
H8—C6—H7	109.5	C16a—C18a—H31A	109.5
C3—C7—C8	109.85 (14)	H29a—C18a—H31A	109.5
C3—C7—C9	109.93 (14)	H30a—C18a—H31A	109.5
C8—C7—C9	111.42 (16)	C18b—C16b—C12	111.3 (3)
C3—C7—H10	108.5	C18b—C16b—C17B	109.5 (5)
C8—C7—H10	108.5	C12—C16b—C17B	108.5 (3)
C9—C7—H10	108.5	C18b—C16b—H25B	109.2
C7—C8—H11	109.5	C12—C16b—H25B	109.2
C7—C8—H12	109.5	C17b—C16b—H25B	109.2
H11—C8—H12	109.5	C16b—C17b—H26B	109.5
C7—C8—H13	109.5	C16b—C17b—H27B	109.5
H11—C8—H13	109.5	H26b—C17b—H27B	109.5
H12—C8—H13	109.5	C16b—C17b—H28B	109.5
C7—C9—H15	109.5	H26b—C17b—H28B	109.5
C7—C9—H14	109.5	H27b—C17b—H28B	109.5
H15—C9—H14	109.5	C16b—C18b—H29B	109.5
C7—C9—H16	109.5	C16b—C18b—H30B	109.5
H15—C9—H16	109.5	H29b—C18b—H30B	109.5
H14—C9—H16	109.5	C16b—C18b—H31B	109.5
N4—C10—C11	111.05 (13)	H29b—C18b—H31B	109.5
N4—C10—C13	121.20 (14)	H30b—C18b—H31B	109.5
C11—C10—C13	127.74 (15)	N3—B—N1	110.66 (12)
C12—C11—C10	105.58 (14)	N3—B—N2^i^	110.67 (12)
C12—C11—H17	127.2	N1—B—N2^i^	108.29 (12)
C10—C11—H17	127.2	N3—B—H1	109.1
N3—C12—C11	107.36 (13)	N1—B—H1	109.1
N3—C12—C16A	123.61 (13)	N2^i^—B—H1	109.1
C11—C12—C16A	129.03 (15)	C3—N1—N2	108.29 (12)
N3—C12—C16B	123.61 (13)	C3—N1—B	125.87 (12)
C11—C12—C16B	129.03 (15)	N2—N1—B	125.66 (11)
C10—C13—C15	111.21 (14)	C1—N2—N1	108.30 (11)
C10—C13—C14	111.11 (15)	C1—N2—B^i^	126.05 (12)
C15—C13—C14	111.14 (17)	N1—N2—B^i^	125.43 (12)
C10—C13—H18	107.7	C12—N3—N4	110.45 (12)
C15—C13—H18	107.7	C12—N3—B	130.79 (12)
C14—C13—H18	107.7	N4—N3—B	118.75 (12)
C13—C14—H20	109.5	C10—N4—N3	105.56 (12)
			
N2—C1—C2—C3	−0.15 (18)	C7—C3—N1—N2	178.58 (14)
C4—C1—C2—C3	−178.52 (15)	C2—C3—N1—B	−175.10 (14)
C1—C2—C3—N1	0.01 (18)	C7—C3—N1—B	3.3 (2)
C1—C2—C3—C7	−178.32 (16)	N3—B—N1—C3	61.64 (19)
N2—C1—C4—C5	−103.02 (17)	N2^i^—B—N1—C3	−176.89 (13)
C2—C1—C4—C5	75.1 (2)	N3—B—N1—N2	−112.79 (14)
N2—C1—C4—C6	134.38 (16)	N2^i^—B—N1—N2	8.7 (2)
C2—C1—C4—C6	−47.5 (2)	C2—C1—N2—N1	0.23 (16)
N1—C3—C7—C8	132.58 (18)	C4—C1—N2—N1	178.73 (13)
C2—C3—C7—C8	−49.3 (2)	C2—C1—N2—B^i^	−174.58 (14)
N1—C3—C7—C9	−104.46 (18)	C4—C1—N2—B^i^	3.9 (2)
C2—C3—C7—C9	73.6 (2)	C3—N1—N2—C1	−0.23 (16)
N4—C10—C11—C12	0.08 (19)	B—N1—N2—C1	175.02 (13)
C13—C10—C11—C12	179.94 (16)	C3—N1—N2—B^i^	174.62 (13)
C10—C11—C12—N3	0.00 (18)	B—N1—N2—B^i^	−10.1 (2)
C10—C11—C12—C16a	−179.54 (16)	C11—C12—N3—N4	−0.08 (17)
C10—C11—C12—C16b	−179.54 (16)	C16a—C12—N3—N4	179.49 (14)
N4—C10—C13—C15	−120.42 (18)	C16b—C12—N3—N4	179.49 (14)
C11—C10—C13—C15	59.7 (2)	C11—C12—N3—B	−178.88 (14)
N4—C10—C13—C14	115.21 (18)	C16a—C12—N3—B	0.7 (2)
C11—C10—C13—C14	−64.6 (2)	C16b—C12—N3—B	0.7 (2)
N3—C12—C16a—C18a	135.3 (3)	N1—B—N3—C12	60.88 (19)
C11—C12—C16a—C18a	−45.3 (4)	N2^i^—B—N3—C12	−59.2 (2)
N3—C12—C16a—C17a	−101.6 (3)	N1—B—N3—N4	−117.84 (14)
C11—C12—C16a—C17a	77.9 (3)	N2^i^—B—N3—N4	122.10 (13)
N3—C12—C16b—C18b	−139.2 (6)	C11—C10—N4—N3	−0.13 (17)
C11—C12—C16b—C18b	40.3 (6)	C13—C10—N4—N3	−179.99 (14)
N3—C12—C16b—C17b	100.3 (4)	C12—N3—N4—C10	0.13 (16)
C11—C12—C16b—C17b	−80.2 (5)	B—N3—N4—C10	179.09 (13)
C2—C3—N1—N2	0.14 (17)		

Symmetry code: (i) −*x*+1/2, −*y*+1/2, −*z*.
